# Impact of the molar activity and PSMA expression level on [^18^F]AlF-PSMA-11 uptake in prostate cancer

**DOI:** 10.1038/s41598-021-02104-6

**Published:** 2021-11-19

**Authors:** Sarah Piron, Jeroen Verhoeven, Emma De Coster, Benedicte Descamps, Ken Kersemans, Leen Pieters, Anne Vral, Christian Vanhove, Filip De Vos

**Affiliations:** 1 Laboratory for Radiopharmacy, Ghent University, Ghent, Belgium; 2 Department of Diagnostic Sciences, Ghent University, Ghent, Belgium; 3 Department of Electronics and Information Systems, IBiTech-MEDISIP, Ghent University, Ghent, Belgium; 4 Department of Medical Imaging, Ghent University Hospital, Ghent, Belgium; 5 Department of Human Structure and Repair, Ghent University, Ghent, Belgium

**Keywords:** Diagnostics, Cancer imaging, Urological cancer

## Abstract

This two-part preclinical study aims to evaluate prostate specific membrane antigen (PSMA) as a valuable target for expression-based imaging applications and to determine changes in target binding in function of varying apparent molar activities (MA_app_) of [^18^F]AlF-PSMA-11. For the evaluation of PSMA expression levels, male NOD/SCID mice bearing prostate cancer (PCa) xenografts of C4-2 (PSMA+++), 22Rv1 (PSMA+) and PC-3 (PSMA−) were administered [^18^F]AlF-PSMA-11 with a medium MA_app_ (20.24 ± 3.22 MBq/nmol). SUV_mean_ and SUV_max_ values were respectively 3.22 and 3.17 times higher for the high versus low PSMA expressing tumors (*p* < 0.0001). To evaluate the effect of varying MA_app_, C4-2 and 22Rv1 xenograft bearing mice underwent additional [^18^F]AlF-PSMA-11 imaging with a high (211.2 ± 38.9 MBq/nmol) and/or low MA_app_ (1.92 ± 0.27 MBq/nmol). SUV values showed a significantly increasing trend with higher MA_app_. Significant changes were found for SUV_mean_ and SUV_max_ between the high versus low MA_app_ and medium versus low MA_app_ (both *p* < 0.05), but not between the high versus medium MA_app_ (*p* = 0.055 and 0.25, respectively). The effect of varying MA_app_ was more pronounced in low expressing tumors and PSMA expressing tissues (e.g. salivary glands and kidneys). Overall, administration of a high MA_app_ increases the detection of low expression tumors while also increasing uptake in PSMA expressing tissues, possibly leading to false positive findings. In radioligand therapy, a medium MA_app_ could reduce radiation exposure to dose-limiting organs with only limited effect on radionuclide accumulation in the tumor.

## Introduction

Prostate specific membrane antigen (PSMA) is a type II transmembrane glycoprotein that exhibits glutamate carboxypeptidase activity in the proximal small intestines (folate hydrolase) and the brain (NAALADase)^1^. Furthermore, PSMA expression can be found on several tissues including prostate tissue, renal tubules, liver, spleen, and salivary and lacrimal glands^2^. In prostate cancer (PCa) cells, PSMA is expressed 100–1000 fold higher. This overexpression is positively correlated with tumor grade, pathological stage and metastatic castration-resistant prostate cancer (mCRPC), making it an attractive target for both diagnostic and therapeutic purposes^3–5^.

For molecular imaging, radiopharmaceuticals are preferably produced with a high molar activity (MA) to ensure that the injected solution contains only a small amount (pico to microgram range) of the tracer substance to avoid pharmacological activity and potential toxic effects. With competitive binding of the radioligand to a target with a saturable receptor density, the chemical mass may affect receptor binding and tracer pharmacokinetics, leading to impaired imaging quality^6^. This mass effect has already been described in the central nervous system, where sub-nanomolar binding affinity ranges and high molar activities are required to distinguish specific from non-specific binding^7,8^. Noguchi et al. demonstrated a positive impact of a high molar activity of [^11^C]raclopride for characterization of the D_2_-receptor in low density regions in the rat brain^9^. For neuroendocrine tumors containing somatostatin receptors, multiple somatostatin-targeting radioligands have shown a dependency between peptide mass and radioligand uptake. Peptide mass-dependent uptake of [^68^Ga]DOTATOC, a radiolabelled somatostatin receptor ligand, was observed both in organs and tumor tissue^10^. ^111^In[DOTA^0^,TYR^3^]octreotide showed an organ-dependent relationship between radioligand uptake and peptide mass, which was suggested to be a balance of the amount of peptide positively influencing receptor clustering while negatively impacting receptor saturation^11^. For [^111^In]pentreotide, a bell-shaped relationship between specific uptake in octreotide receptor-positive tissues and carrier dose was found. This introduced an additional parameter to ameliorate the target-to-background contrast. Attributing factors to this phenomenon could be receptor accessibility (blood perfusion, presence of endogenous ligand) and receptor binding properties (dissociation constant, rate of internalization and re-expression rate of the receptor)^12^. Furthermore, a simulation study using PBPK modelling investigated the effect of ligand amount, affinity and internalization rate on PSMA imaging and RLT. Overall, the influence of the amount of ligand was more pronounced for radiotracers with a dissociation constant < 1 nM as well as for therapy compared to imaging. Increasing ligand amount significantly reduced absorbed doses in highly perfused or high affinity tissues^13^. These studies show that the amount of peptide administered could be optimized to increase absolute tumor uptake and tumor-to-background ratios.

The impact of the molar activity as a parameter for PET imaging has been investigated for several imaging targets, and it was reported to be highly pronounced for radioligands targeting PSMA. Increasing the amount of peptide had predominantly an effect on uptake in PSMA expressing organs compared to tumors which seemed to be tissue-dependent^14^. Soeda et al. also demonstrated a higher sensitivity of the salivary glands to increasing amounts of peptide compared to tumor tissue using [^18^F]PSMA-1007, potentially minimizing xerostomia during PSMA radioligand therapy (RLT)^15^. In this two-part preclinical study, we aim to validate PSMA as a target for expression-based imaging by determining the relationship between differences in PSMA expression levels and tumor uptake. Furthermore, changes in the molar activity/amount of peptide of [^18^F]AlF-PSMA-11 on uptake parameters and tumor-to-organ ratios will be evaluated.

## Materials and methods

### Synthesis of [^18^F]AlF-PSMA-11

[^18^F]AlF-PSMA-11 was synthesized on a modified SynthraFCHOL synthesis module (Synthra GmbH, Hamburg, Germany) as previously reported with some minor modifications^16^. The radiochemical purity was 95.9–97.1% as determined by thin layer chromatography (Alugram RP18-W/UV254 plates (Machery Nagel, Düren, Germany)) using 3:1 v/v acetonitrile in water as mobile phase (Supplementary Fig. 1). Molar activities between 76 and 538 MBq/nmol were achieved as analysed by high performance liquid chromatography (Prevail C18 reversed-phase column, 4.6 × 250 mm, 5 µm, Lokeren, Belgium) and a calibrated dose calibrator (Biodex medical systems, USA) (Supplementary Fig. 2). To achieve differences in apparent molar activity (MA_app_) levels, varying amounts of a 0.1 µg/µL PSMA-11 (ABX) solution were added to a stock vial to obtain solutions of 1.92 ± 0.27 MBq/nmol (low MA_app_), 20.06 ± 3.22 MBq/nmol (medium MA_app_) and 211.2 ± 38.9 MBq/nmol (high MA_app_).

### Preparation of tumor models

The study was approved by the Ghent University Ethical Committee on animal experiments (ECD 18/116). All animals were kept and handled according to the European guidelines (Directive 2010/63/EU). PCa cell lines C4-2 (ATCC; CRL-3314), 22Rv1 (ATCC; CRL-2505) and PC-3 (ATCC; CRL-1435) were cultured using RPMI 1640 medium supplemented with 10% FBS, 1% streptomycin/penicillin (10,000 U/mL) and 1% glutamine 200 mM, and maintained at 37 °C in 5% CO_2_ in humidified air. For inoculation, PCa cells were rinsed with FBS-free RPMI 1640 medium and cell suspensions of 5 × 10^6^ cells/100 µL were prepared. Four-to-six-week-old male NOD/SCID mice (Janvier, France) were subcutaneously injected at shoulder height with 200 µL 1:1 cell suspension:Matrigel on either side of each mouse (C4-2, n = 8; 22Rv1, n = 6; PC-3, n = 5). Tumor growth was monitored weekly for 5–6 weeks until tumors reached a diameter between 5 and 10 mm.

### Small animal PET/CT imaging

To determine the impact of varying molar activities, each C4-2 xenograft bearing mouse underwent three PET/CT scans within a timeframe of 14 days. On day 1, 7 and 14, mice received either high/low/medium MA_app_ (n = 2) or medium/low/high MA_app_ (n = 4), respectively. The mean administered [^18^F]AlF-PSMA-11 activity was 9.29 ± 0.37 MBq with a mean MA_app_ of either 196.8 ± 32.4 MBq/nmol (high MA_app_), 19.10 ± 1.69 MBq/nmol (medium MA_app_) or 1.94 ± 0.274 MBq/nmol (low MA_app_). To evaluate the activity uptake in PCa tumors with different PSMA expression levels, the images of the medium MA_app_ of C4-2 were compared to PC-3 and 22Rv1. The mean administered activity was 9.30 ± 0.62 MBq [^18^F]AlF-PSMA-11 with a mean medium MA_app_ of 20.06 ± 3.22 MBq/µg. To evaluate whether the influence of the MA_app_ on low expression tumors, 22Rv1 xenograft bearing mice underwent one additional PET/CT scan on day 7. Each mouse was administered 9.47 ± 0.24 MBq [^18^F]AlF-PSMA-11 with a high MA_app_ of 243.8 ± 32.8 MBq/nmol.

The images were acquired in list mode using a small animal PET scanner (β-cube, Molecubes, Ghent, Belgium) with a spatial resolution of 0.85 mm and an axial field-of-view of 13 cm. All PET scans were reconstructed into a 192 × 192 × 384 matrix by an ordered subsets maximization expectation (OSEM) algorithm using 30 iterations and a voxel size of 400 × 400 × 400 µm^3^.

### Immunohistochemical evaluation

After the last scan, two mice/cell line xenograft were sacrificed and tumors were collected for immunohistochemical (IHC) evaluation. Sections were taken from the centre of the tumor sample and stained using Haematoxylin/Eosin, incubated with a primary PSMA antibody (1:400, 2 h, ab133579, Abcam) and counterstained using Haematoxylin (Mayer). Sections were digitally scanned with a virtual scanning microscope (Olympus BX51, Olympus Belgium SA/NV, Berchem, Belgium) at high resolution (20 × magnification).

### Western blot analysis

The tumors of the other mice were harvested for western blot (WB) analysis to quantify the PSMA expression levels of the tumor tissues. After thorough rinsing of the tumors, tissue lysates were prepared using RIPA sample buffer. Protein concentrations were determined using the Pierce BCA Protein assay (ThermoFisher Scientific). Samples containing 50 µg of total protein were loaded on 8.5% sodium dodecyl sulphate–polyacrylamide gel electrophoresis (SDS-PAGE) gels. After the transfer, the nitrocellulose membranes were blocked using Odyssey Blocking Buffer (Li-cor Biosciences, Bad Homburg, Germany). Membranes were incubated overnight using a monoclonal PSMA Rabbit anti-Human primary antibody (1:2000, MA533086, Invitrogen, Fischer Scientific, Belgium) and polyclonal Rabbit anti-Human beta actin primary antibody (1:1000, PA1-183, Invitrogen, Fischer Scientific, Belgium), followed by 1 h incubation with IRDye® 800CW Goat anti-Rabbit IgG secondary antibody (1:15,000, Li-cor Biosciences, Bad Homburg, Germany) (Supplementary Fig. 3). The relative densities of both PSMA and β-actin bands were determined using ImageJ^17^.

### Data analysis

Co-registration and analysis of the PET/CT images was performed using PMOD (PMOD Technologies®, Zürich, Switzerland). Images were visually analysed using the PROMISE criteria. This standardizes image interpretation based on activity uptake in reference tissues (blood, liver and salivary gland) and subdivides suspicious lesions into no, low, intermediate or high PSMA expression^18^. Volumes of interest (VOIs) were drawn manually for delineating the tumor, kidneys, bladder, salivary and lacrimal glands, heart (blood), liver, spleen, muscle and bone. Tissue uptake in each VOI was corrected for residual activity in the syringe and radioactive decay and expressed as SUV_mean_ and SUV_max_. Furthermore, tumor-to-organ ratios of tissues with a high likelihood for metastases including tumor-to-liver (TLR), tumor-to-muscle (TMR) and tumor-to-bone ratio (TBoR) were determined.

### Statistical analysis

All uptake parameters (SUV_mean_, SUV_max_, TLR, TMR and TBoR) were reported as mean ± SD. The statistical analysis was performed in R^19^ using the Mann–Whitney U test for comparison of uptake between different PSMA expressing xenografts and the Kruskal Wallis test for comparing radioligand uptake of varying MA_app_, followed by post-hoc pairwise comparison using the Wilcoxon-signed rank test with holm correction for multiple testing. The significance level was set on *p* ≤ 0.05.

### Ethics approval and consent to participate

The study was approved by the Ghent University Ethical Committee on animal experiments (ECD 18/116). All animals were kept and handled according to the European guidelines (Directive 2010/63/EU). The manuscript complies with the ARRIVE guidelines.

## Results

### Semi-quantitative analysis of PSMA expression levels in prostate cancer cell lines

**Figure 1:**
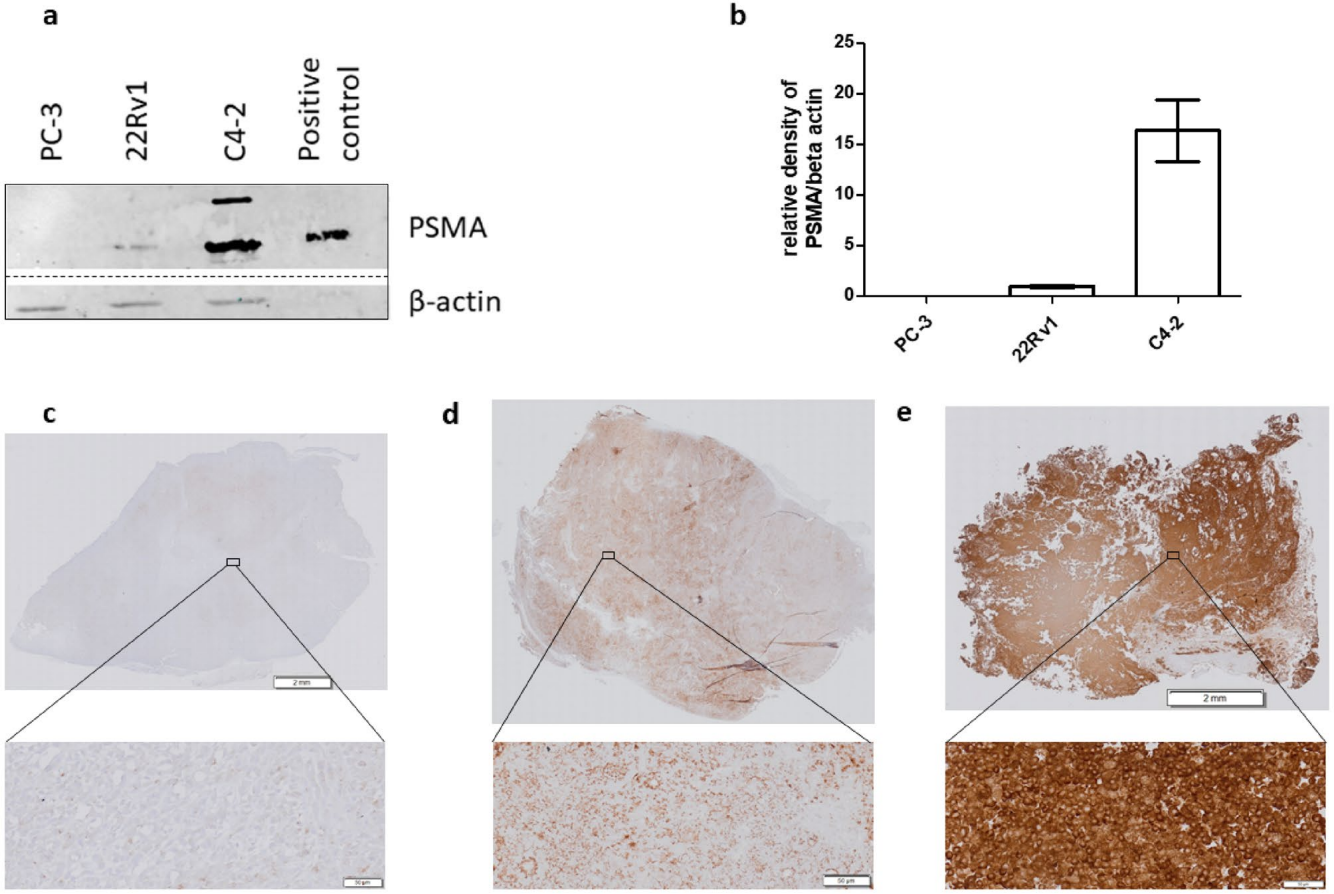
Analysis of PSMA expression levels. WB of PCa tumor tissues and a positive control, all samples were run on the same blot. The non-relevant bands were cropped from the image, the full-length blot is presented in Supplementary Fig. 1 (**a**). Semi-quantification of WB, the relative density of PSMA was normalized using β-actin as loading control. Quantification was only performed using bands from the same blot (**b**). Data for PC-3, 22Rv1 and C4-2 (n = 5) is presented as mean ± SD. IHC of PSMA expression of PC-3 (**c**), 22Rv1 (**d**) and C4-2 (**e**) tumor tissue.

The PSMA expression levels were determined by both western blot and immunohistochemical assessment (Fig. 1). WB analysis detected the highest presence of PSMA expression in C4-2 tissue, while only moderate to low expression levels could be observed for 22Rv1. Semi-quantification of blots revealed a β-actin normalized density for PSMA expression of 16.36 ± 3.05 for C4-2 and 0.94 ± 0.21 for 22Rv1 tumors. PC-3 tumors did not show PSMA expression. Similar results were demonstrated by IHC staining, where all C4-2 cells were clearly positively stained for PSMA, while 22Rv1 showed weaker and more heterogeneous staining of the tumor tissue.

### Relationship between PSMA expression levels and [^18^F]AlF-PSMA-11 tumor uptake

**Figure 2:**
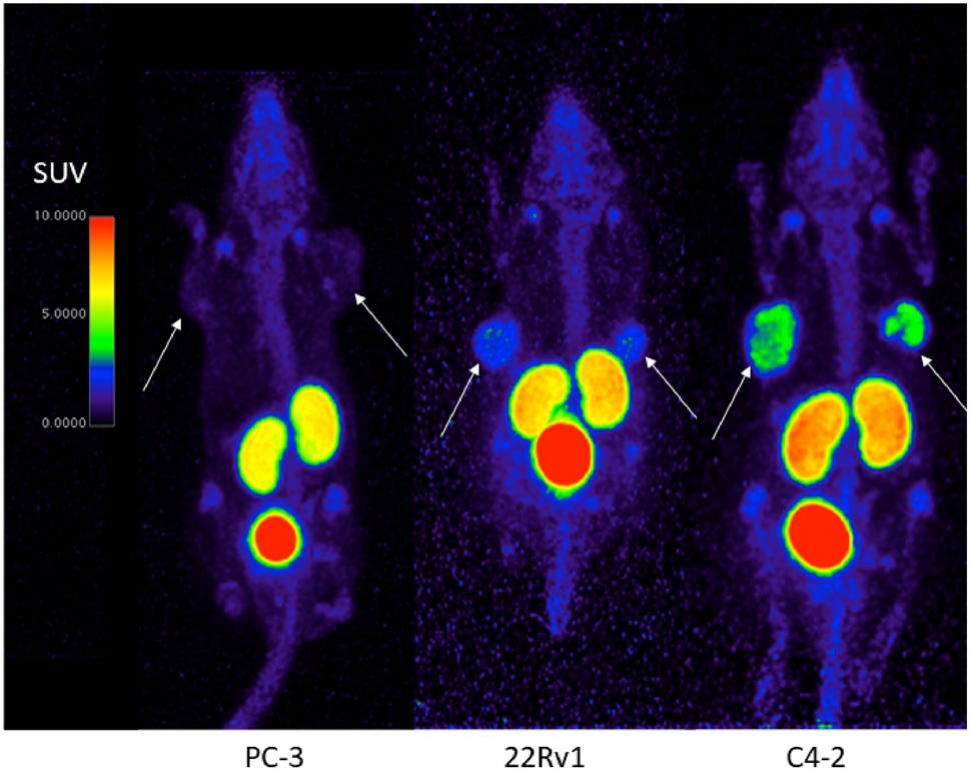
Representative PET images of PC-3 (left), s22Rv1 (middle) and C4-2 (right) xenografts 60 min p.i. of [^18^F]AlF-PSMA-11 with medium MA_app_. Arrows indicate tumors. There was no activity uptake detectable in PC-3 tumors, C4-2 and 22Rv1 tumors were visible, although the latter showed less tumor contrast. Color maps were generated using Horos v4.0.0, https://horosproject.org/.

Comparative images of [^18^F]AlF-PSMA-11 uptake (medium MA_app_) in different PSMA expressing tumors are presented in Fig. 2. Tumor uptake was positively correlated with higher expression levels. According to the PROMISE criteria, PC-3 tumors were PSMA negative (score 0) as tumor uptake was comparable with activity in the blood (heart). 22Rv1 tumors were visually detectable and the tumor activity was higher than the liver and salivary glands, which corresponds to high PSMA expression (score 3). C4-2 tumors showed good image contrast with tumor uptake above activity in the salivary glands (score 3) (Supplementary Table 1).

Quantitative VOI analysis demonstrated significant differences for SUV_mean_ and SUV_max_ as well as tumor-to-organ ratios including tumor-to-liver, tumor-to-blood and tumor-to-muscle in function of PSMA expression levels (Table 1). When comparing C4-2 (high PSMA expression) to 22Rv1 (low PSMA expression), SUV_mean_ and SUV_max_ values were approximately 3.22 and 3.17 times higher, respectively (*p* < 0.0001). Similar ratios of 2.77, 2.23 and 2.96 were found for tumor-to-liver, tumor-to-blood and tumor-to-muscle, respectively (*p* < 0.0001). SUV values were significantly lower for PC-3 tumors compared to both C4-2 (*p* < 0.01) and 22Rv1 tumors (*p* < 0.001).

**Table 1:** SUV values and tumor-to-organ ratios (liver, blood and muscle) for different PCa tumor cell lines with varying PSMA expression levels: PC-3 (no PSMA expression), 22Rv1 (low PSMA expression) and C4-2 (high PSMA expression).

Cell line	Expression level^a^	SUVmean	SUVmax	Tumor-to-liver ratio	Tumor-to-blood ratio^b^	Tumor-to-muscle ratio
PC-3	0	0.09 ± 0.02**	0.22 ± 0.05**	1.38 ± 0.33**	1.41 ± 0.07**	2.26 ± 0.48**
22Rv1	0.94 ± 0.21	0.46 ± 0.11****	1.03 ± 0.23****	6.26 ± 1.41****	6.93 ± 1.49****	6.64 ± 3.81****
C4-2	16.36 ± 3.05	1.48 ± 0.47	3.27 ± 1.07	17.32 ± 3.55	15.47 ± 2.32	19.64 ± 5.37

*P* values were calculated using the Wilcoxon signed-rank test with Holm correction with C4-2 as a reference, the significance level was set at *p* = 0.05, **p < 0.01, ****p < 0.0001.

^a^Expression levels were determined by semi-quantitative western blot analysis and are expressed β-actin normalized density.

^b^Activity in blood was determined by delineation of the heart.

### Impact of the molar activity on tumor uptake

**Figure 3:**
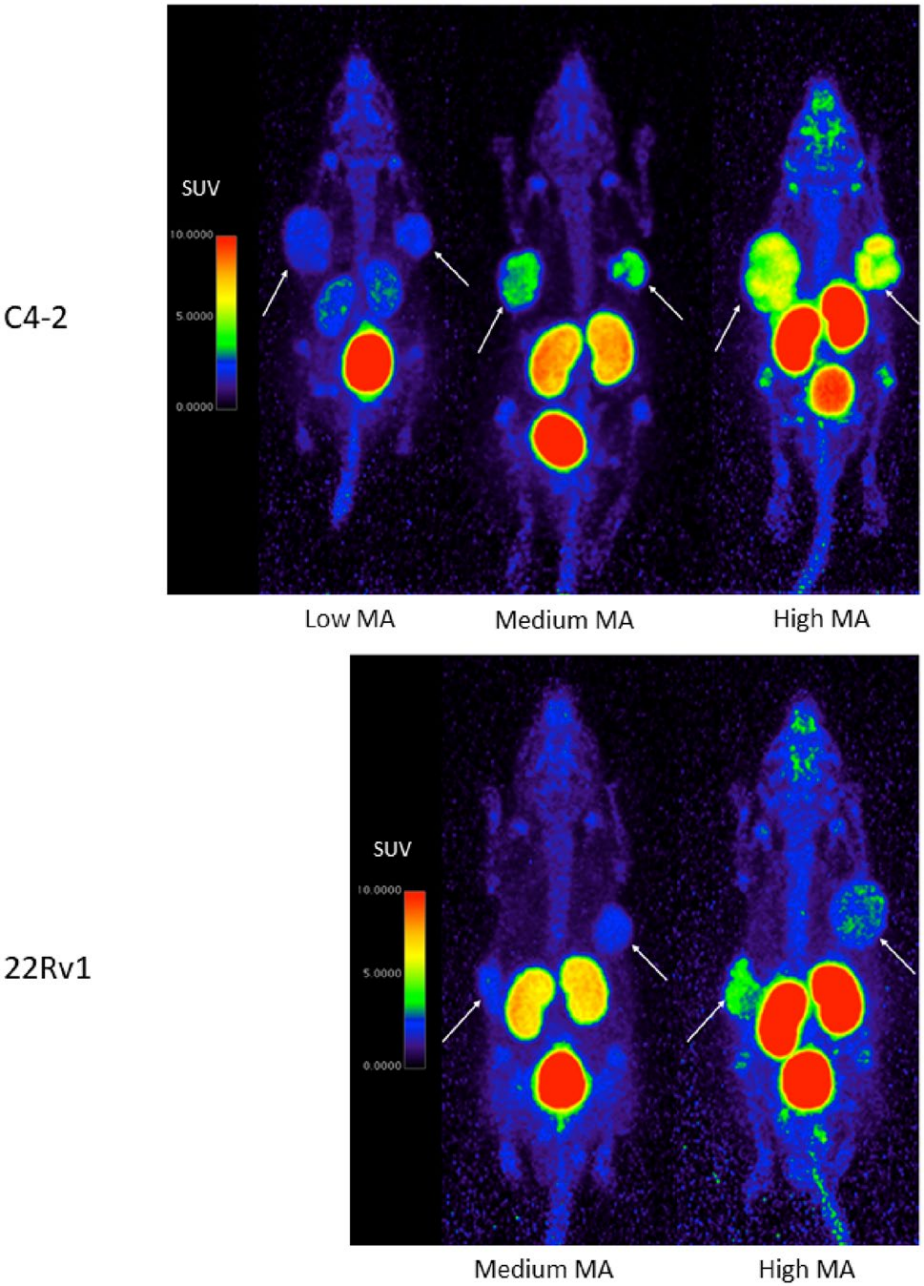
Representative PET images after [^18^F]AlF-PSMA-11 injection of C4-2 (top) and 22Rv1 (bottom) xenografts of low (only C4-2), medium and high MA_app_ images in the same animal. Arrows indicate tumors. Increasing MA_app_ resulted in higher activity uptake in tumors as well as the kidneys, while activity in the bladder decreased. Color maps were generated using Horos v4.0.0, https://horosproject.org/.

To evaluate the impact of the MA_app_ on tumor uptake, all C4-2 xenograft bearing mice underwent three PET/CT scans with different MA_app_ levels (high MA_app_ = 194.8 ± 32.1 MBq/nmol, medium MA_app_ = 18.91 ± 1.67 MBq/nmol and low MA_app_ = 1.92 ± 0.27 MBq/nmol). Images show high uptake in C4-2 tumors after injection of a high MA_app_ activity dose, which decreased with lower MA_app_ (Fig. 3). Kidney uptake decreased as well with lower MA_app_ while activity in de bladder increased. PSMA expressing tissues such as salivary glands, lacrimal glands and spleen were clearly visible on high MA_app_ PET images but were barely visible on medium MA_app_ images and not detectable on low MA_app_ images (Supplementary Table 2).

**Figure 4:**
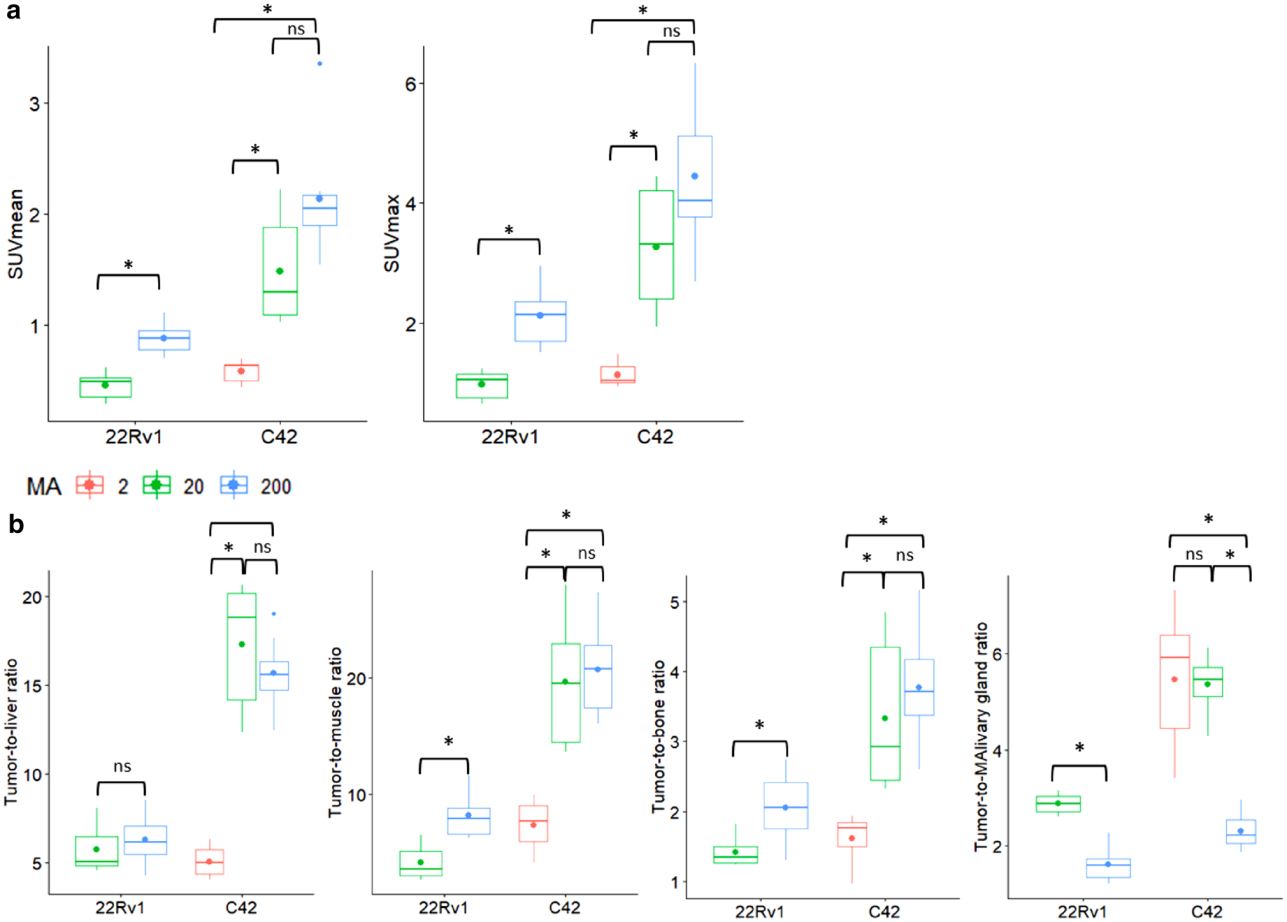
SUV_mean_ (left) and SUV_max_ (right) (**a**) and tumor-to-liver, tumor-to-muscle, tumor-to-bone and tumor-to-salivary gland ratio (**b**) of 22Rv1 and C4-2 tumors after imaging with either the low MA_app_ (0.94 ± 0.27 MBq/µg, only C4-2), medium MA_app_ (20.26 ± 3.25 MBq/µg) or high MA_app_ (213.3 ± 39.3 MBq/µg) [^18^F]AlF-PSMA-11 60 min p.i.. The mean values were added as dots to the boxplot. The significance level was set at *p* = 0.05. *Ns* = not significant, *p < 0.05.

When comparing intra-individual tumor uptake of the high, medium and low MA_app_, SUV_mean_ (2.14 ± 0.54, 1.48 ± 0.47 and 0.58 ± 0.10, respectively) and SUV_max_ (4.44 ± 1.25, 3.27 ± 1.07 and 1.15 ± 0.19, respectively) showed a significantly decreasing trend (both *p* < 0.001). Post-hoc pairwise comparison revealed statistically significant changes between the high and low MA_app_ (*p* < 0.05) as well as between the medium and low MA_app_ (*p* < 0.05), but not between the high and medium MA_app_ (*p* = 0.055 and 0.25, respectively) (Fig. 4a). Additionally, 22Rv1 xenograft bearing mice (n = 3) received an additional PET/CT scan with high MA_app_ (246.3 ± 33.1 MBq/µg). Comparison of SUV_mean_ and SUV_max_ values demonstrated a statistically significant difference between the high and medium MA_app_ (both *p* = 0.031).

**Figure 5:**
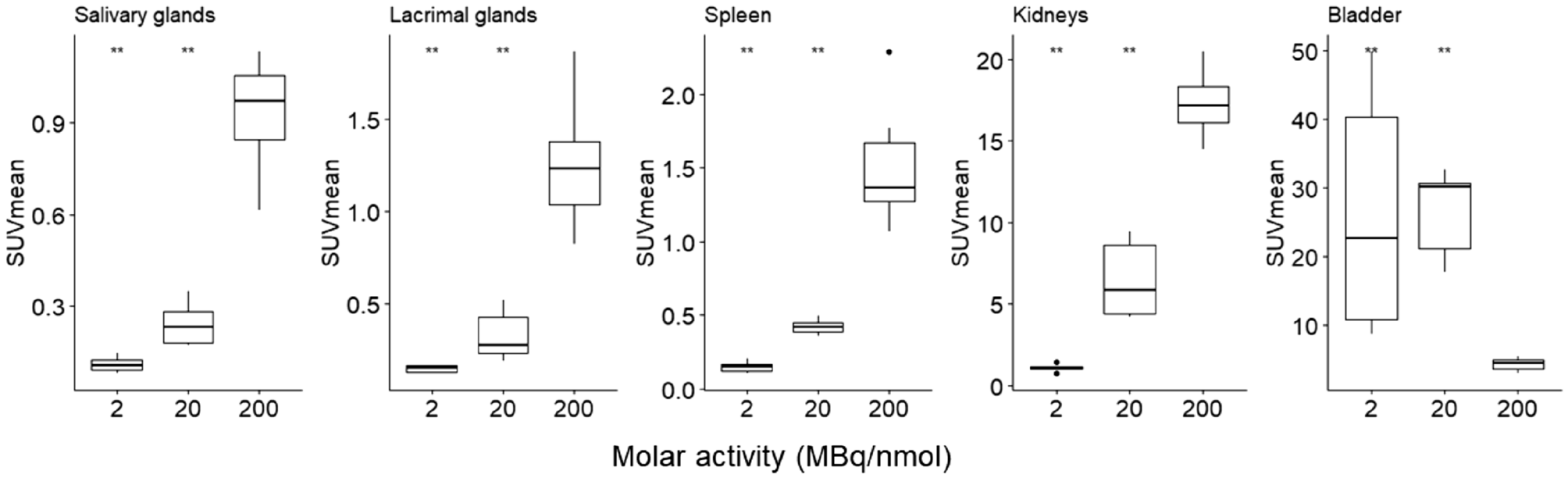
C4-2 xenograft bearing mice were injected with [^18^F]AlF-PSMA-11 with either a low MA_app_ (1.92 ± 0.27 MBq/µg), medium MA_app_ (18.91 ± 1.67 MBq/µg) or high MA_app_ (194.8 ± 32.1 MBq/µg). Boxplots represent SUV_mean_ values 60 min p.i. of PSMA expressing tissues including salivary glands, lacrimal glands, spleen and excretory organs (kidneys and bladder). The high MA_app_ was set as reference and the significance level was set at *p* = 0.05. *Ns* = not significant, **p < 0.01.

Tumor-to-organ ratios were determined for tissues with a high likelihood for metastases including liver (TLR), muscle (lymph nodes) (TMR) and bone (TBoR). In C4-2 xenografts, tumor-to-organ ratios remained constant between the high and medium MA_app_ for TLR (15.71 ± 1.99 vs 17.32 ± 3.55, *p* = 0.46), TMR (20.69 ± 3.82 vs 19.64 ± 5.37, *p* = 0.74) and TBoR (3.77 ± 0.78 vs 3.33 ± 1.03, *p* = 0.31) while a statistically significant difference could be observed for high versus low MA_app_ and medium versus low MA_app_ (Fig. 4b). A similar trend between the high and medium MA_app_ was found for 22Rv1 xenografts, however, the tumor-to-liver ratio remained constant. On the other hand, SUV_mean_ values of PSMA expressing tissues such as salivary and lacrimal glands, spleen, and kidneys significantly decreased between the high and medium MA_app_ (*p* < 0.01) (Fig. 5). Tumor-to-salivary gland ratios increased from 2.30 ± 0.37 for the high MA_app_ to 5.35 ± 0.59 for the medium MA_app_. While kidney uptake significantly decreased, uptake in the bladder rose for the medium and low MA_app_.

## Discussion

Although the diagnostic applications of PSMA as a target for imaging have been the subject of many clinical trials, preclinical studies are essential for a comprehensive investigation of several important aspects of PSMA PET imaging. In this paper, [^18^F]AlF-PSMA-11 was used for imaging experiments to further understand PSMA PET imaging. First, PSMA was validated as a valuable target for expression-based applications by determining the relationship between PSMA expression levels and [^18^F]AlF-PSMA-11 tumor uptake. This was examined using three human PCa cell lines with high (C4-2), low (22Rv1) and no detectable (PC-3) PSMA expression. Second, changes in target binding of PSMA positive tumors and tissues using varying amounts of cold PSMA-11 peptide were evaluated.

The relationship between target expression levels and [^18^F]AlF-PSMA-11 uptake was determined by PET/CT imaging of mice bearing PCa tumors with varying PSMA expression levels. The use of a medium MA_app_ for the expression level experiments was selected based on several practical considerations. A larger group of mice could be imaged on the same day, while the use of the high MA_app_ would significantly limit the size of the study population. Also, using the high MA_app_ would introduce a larger variation in the MA_app_ between animals, as the amount of PSMA-11 precursor is so small, that minor differences in amount of precursor added to the stock solution would lead to large differences in MA_app_. Semi-quantitative western blot analysis showed that PSMA expression levels of C4-2 tumors were approximately 16 times higher compared to 22Rv1 tumors, while SUV values were only 3 times higher. A positive association between PSMA expression and SUV values could be found. Although 22Rv1 tumors had only low PSMA expression levels on western blot, activity uptake was above the liver, scoring positively (score 3) according to the PROMISE criteria. This suggests the ability of [^18^F]AlF-PSMA-11 to detect PCa lesions, even with low PSMA receptor abundancy. Increasing the MA_app_ of [^18^F]AlF-PSMA-11 increased absolute SUV_mean_ and SUV_max_ values (Fig. 4), but due to higher salivary gland uptake, the PROMISE score did not increase. However, TMR and TBoR did increase significantly, which could be beneficial for the detection of lymph nodes and bone metastases with low PSMA expression levels, although this should be confirmed in a murine model of bone metastases. Lückerath et al. determined the in vivo correlation between [^68^Ga]PSMA-11 PET/CT and PSMA expression using four murine PCa cell lines. [^68^Ga]PSMA-11 PET was able to detect changes in PSMA expression at low levels, but this sensitivity was lost at higher PSMA levels, which is in agreement with the reported non-linear correlation^20^.

PSMA expression was shown to be an important prognosticator for overall survival of metastatic castrate resistant prostate cancer (mCRPC) patients under [^177^Lu]PSMA treatment. The presence of low average-PSMA expressing tumors was found to be a negative prognostic factor in these patients^21^. The threshold for determining the PSMA expression level was based on the criterium set by Hofman et al. who defined high PSMA expression as any metastatic lesion with a SUV_max_ of 1.5 times the liver SUV. Here, [^177^Lu]PSMA therapy resulted in 50% decline in PSA for 17/30 mCRPC patients presenting with high PSMA expression metastases^22^. The liver seems to be a solid choice as reference organ as our results show that changes in the MA_app_ did not significantly alter tumor-to-liver ratios (between the high and medium MA_app_). It should however be noted that the difference in chemical structure between [^177^Lu]PSMA-617 and [^18^F]AlF-PSMA-11 could also have an influence on biodistribution and clearance pathways. Additional research should determine to which extent PSMA expression levels could be used as an exclusion criterium for [^177^Lu]PSMA therapy. In our study, the difference between the medium and high MA_app_ was more pronounced in low expression 22Rv1 tumors. SUV values, TMR and TBoR increased significantly when administering the high MA_app_. This can be attributed to the lower amount of receptors available to bind radiolabelled PSMA molecules and more rapid saturation in the presence of a larger peptide mass.

Overall, the highest mean absolute SUV_mean_ and SUV_max_ values were obtained after administration of [^18^F]AlF-PSMA-11 with high MA_app_, but the difference between the medium and high MA_app_ was not statistically significant, both for SUV values as for tumor-to-organ ratios (TLR, TMR and TBoR). There was however a substantial decrease of activity uptake in PSMA expressing tissues (salivary and lacrimal glands, spleen and kidneys) between the high and medium MA_app_. The use of medium MA_app_ [^18^F]AlF-PSMA-11 could therefore reduce activity uptake in PSMA positive healthy tissues while maintaining image contrast of tumor metastases compared to the surrounding tissue. This would be beneficial for both diagnostic purposes and radioligand therapy. Prostate cancer lesions could be more reliably detected due to the constant tumor-to-organ ratio while less non-specific uptake could facilitate scan interpretation by limiting the potential pitfalls of PSMA PET caused by aspecific physiological uptake^23^. In radioligand therapy, healthy tissue would be less exposed to the radiation while absolute tumor uptake would remain approximately constant.

During PSMA radioligand therapy, radiation exposure caused by activity uptake in PSMA-expressing non-tumor tissues can cause dose-limiting toxic side effects. Radioligand uptake in the salivary glands causes xerostomia and can be a reason for treatment discontinuation^24,25^. Lowering the MA_app_ for RLT ligands could be a useful tool for reducing radiation exposure and should be further investigated as an alternative for botulinum toxin injections^26^ or sialendoscopy and dilatation combination with saline irrigation and steroid injections^27^. A preclinical study by Fendler et al. also showed that administration of [^177^Lu]PSMA-617 with a molar activity of 62 MBq/nmol achieved the highest tumor-to-salivary gland ratio compared to lower molar activities (31 and 15 MBq/nmol)^28^. When correcting for the administered dose of 60 MBq, the highest molar activity corresponds to 0.97 nmol PSMA-617, which is in the same range as our medium MA_app_ group (0.49 nmol PSMA-11) that also showed the highest tumor-to-salivary gland ratio. However, it was reported that the low to moderate PSMA staining on IHC staining does not correlate with the high PSMA radioligand accumulation. It was hypothesized that tracer accumulation in the salivary glands is therefore a combination of both specific and non-specific uptake^29^. The latter mechanism is not yet elucidated and our results show a large impact of a higher amount of peptide on activity uptake in salivary glands, suggesting that this as a promising direction for further research. The difference in PSMA expression between murine and human salivary glands must also be taken into consideration. As the binding affinity and PSMA concentration in murine salivary glands is lower compared to human salivary glands, it would be expected that the influence of molar activities will be more pronounced in humans^30^. Another organ-at-risk are the kidneys. Renal clearance can lead to accumulation of radiolabelled peptides in the tubular cells where it irradiates the kidneys, potentially leading to renal dysfunction, particularly in patients with a known history of nephropathy. Although no major nephrotoxicity was reported for [^177^Lu]PSMA^31^, chronic kidney disease was reported in two patients for [^225^Ac]PSMA therapy. Limiting kidney exposure to [^255^Ac]PSMA may decrease toxicity and maintain its therapeutic potential^32^. A study by Kratochwil et al. investigated the influence of additional 2-PMPA administration on tumor and kidney uptake using [^125^I]MIP-1095. Injection of low doses 2-PMPA (0.2–1 mg/kg) 16 h after tracer administration significantly increased the tumor-to-kidney ratio^33^. These results indicate potential benefits of the use of cold precursor or PSMA inhibitory small molecules such as 2-PMPA for reducing radiation exposure to the kidneys. Additionally, a high tumor load in patients can also lead to reduced activity uptake in dose-limiting organs^34^. [^18^F]AlF-PSMA-11 might therefore be applied as a diagnostic tool for individual optimization of RLT protocols considering PET-based tumor load and radioligand MA_app_.

These results show the importance of the amount of carrier on tumor targeting strategies. The optimal amount should be low to avoid competitive binding of unlabelled molecules at the target site but not too low as the tracer molecules could be trapped in non-specific binding places. For diagnostic purposed, using a high MA_app_ will increase the detection of low expression tumors while also increasing specific uptake in PSMA expressing tissues, possibly leading to false positive findings. For radioligand therapy, further research should focus on finding a balance between administration of a high MA_app_ for high absolute uptake in the tumor tissue while minimizing activity uptake in healthy organs in order to reduce dose-limiting toxicity. Also, although the considerations mentioned before are valid for high PSMA expressing PCa lesions, decreasing the MA_app_ to limit non-specific uptake could also affect the detectability of low PSMA expressing lesions, which could lead to an underestimation of the tumor burden.

A major limitation of this study is the translation from mouse to human. Although the differences in the administered MA_app_ in mice gave well defined results, the activity dose and amount of peptide that is administered compared to total body weight for PET/CT is different for mice and humans, which makes translation into clinical applications more challenging. However, as the molar activity of a PSMA PET tracer decreases over time, it would be beneficial to evaluate the effect on both tumor uptake and non-target uptake in humans. In a retrospective analysis of patients who underwent [^18^F]rhPSMA-7.3 PET/CT, only minor effects on biodistribution were found for a tenfold decrease of MA_app_, except for the salivary glands and spleen, which showed significantly lower uptake for the lower molar activity^35^. Overall, these results encourage a prospective clinical trial using [^18^F]AlF-PSMA-11 comparing a high to medium MA_app_.

Due to the intra-individual comparison of MA_app_ levels, most mice were imaged multiple times and tumor sizes differed slightly between scans. However, the tumor size was many times higher than the PET resolution and mice were randomized for MA_app_ sequence, therefore minimizing this limitation as much as possible.

## Conclusion

This paper evaluated [^18^F]AlF-PSMA-11 PET for expression-based imaging of prostate cancer and the effect of the amount of carrier administered on tumor uptake and tumor-to-organ ratios. A positive association was found between PSMA expression and tumor uptake. The highest absolute tumor uptake was obtained by the high MA_app_. And although activity uptake in PSMA expressing organs decreased significantly with lower MA_app_, there was no statistically significant difference in tumor SUV between the high and medium MA_app_. These results suggest that administration of a high MA_app_ increases the detection of low expression tumors while also increasing specific uptake in PSMA expressing tissues, possibly leading to false positive findings. In radioligand therapy, a medium MA_app_ could reduce radiation exposure to dose-limiting organs with only limited effect on radionuclide accumulation in the tumor. Overall, it is of utmost importance to validate the association between both PSMA expression and molar activity relative to radioligand activity uptake.

## Supplementary Information

Supplementary Information.

## Data Availability

The datasets and images used and/or analysed during the current study are available from the corresponding author on reasonable request.

## Abbreviations

CT: Computed tomography
IHC: Immunohistochemistry
mCRPC: Metastatic castrate resistant prostate cancer
NAALADase: *N*-Acetylated-α-linked-acidic dipeptidase
OSEM: Ordered subsets maximization expectations
PCa: Prostate cancer
PET: Positron emission tomography
PSMA: Prostate specific membrane antigen
RLT: Radioligand therapy
MA_app_: Apparent molar activity
SDS-PAGE: Sodium dodecyl sulphate–polyacrylamide gel electrophoresis
SUV: Standardized uptake value
TBoR: Tumor-to-bone ratio
TLR: Tumor-to-liver ratio
TMR: Tumor-to-muscle ratio
VOI: Volume of interest
WB: Western blot
