# Supplementary material for: Impact of the molar activity and PSMA expression level on [18F]AlF-PSMA-11 uptake in prostate cancer
Source: Sci Rep. 2021 Nov 19;11:22623. doi: 10.1038/s41598-021-02104-6 (PMC8604919; doi:10.1038/s41598-021-02104-6)
Supplement: Supplementary file 1 — Supplementary Information. [file 41598_2021_2104_MOESM1_ESM.docx]

Supplementary Data

Suppl Figure 1: Representative image of thin layer chromatography of [^18^F]AlF-PSMA-11 synthesis.


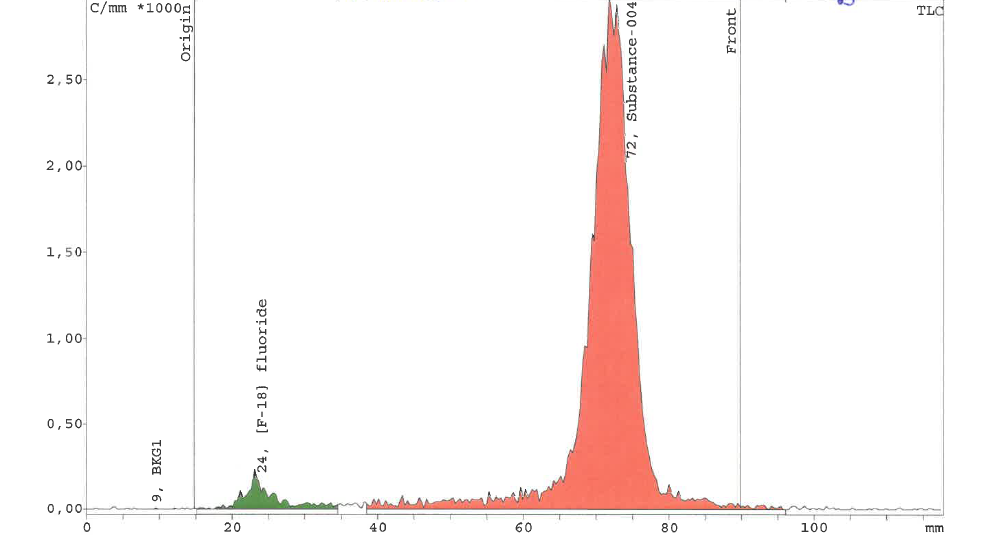


*
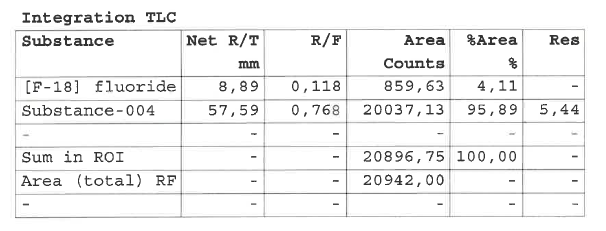
*

Suppl Figure 2: Representative image of high performance liquid chromatography of [^18^F]AlF-PSMA-11 synthesis.

*
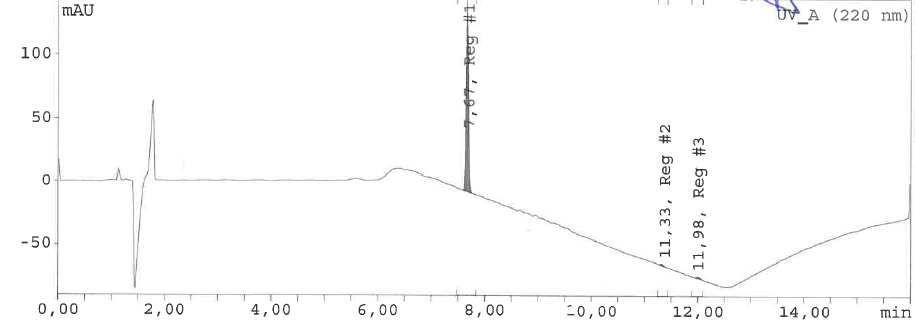
*

*
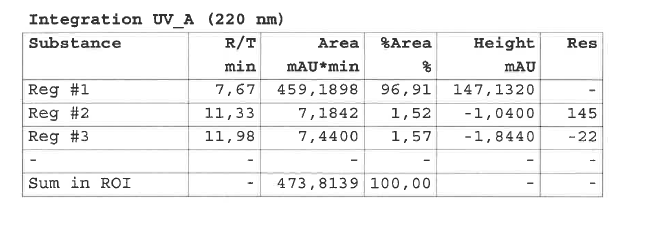
*

Suppl Figure 3: Full length blot of PSMA expression levels.


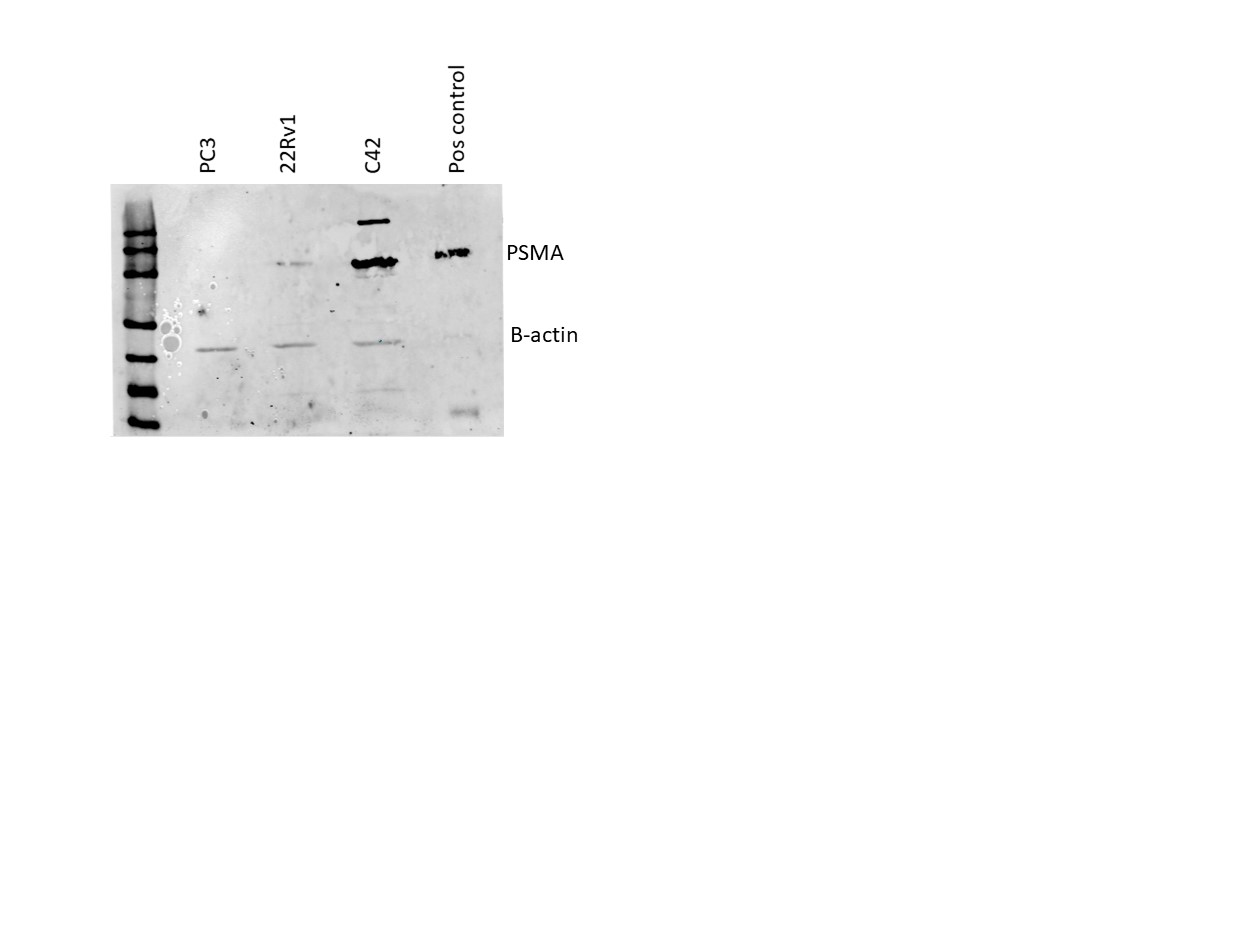


Suppl Table 1: Summary of SUV_mean_ values of C4-2, 22Rv1 and PC-3 xenograft bearing mice for the medium MA_app_.

|  | C4-2 | 22Rv1 | PC-3 |
| --- | --- | --- | --- |
| Blood (Heart) | 0.10 ± 0.04 | 0.07 ± 0.02 | 0.06 ± 0.02 |
| Liver | 0.09 ± 0.03 | 0.07 ± 0.01 | 0.07 ± 0.02 |
| Salivary glands | 0.24 ± 0.07 | 0.15 ± 0.04 | 0.14 ± 0.03 |
| Tumor | 1.48 ± 0.47 | 0.46 ± 0.11 | 0.09 ± 0.02 |
| PROMISE criterium | High expression | High expression | No expression |
| Score | 3 | 3 | 0 |

Suppl Table 2: Summary of SUV_mean_ and SUV_max_ values of C4-2 xenograft bearing mice for the low, medium and high MA_app_.

| Organ | Low MA_app_ | | Medium MA_app_ | | High MA_app_ | |
| --- | --- | --- | --- | --- | --- | --- |
|  | SUV_mean_ | SUV_max_ | SUV_mean_ | SUV_max_ | SUV_mean_ | SUV_max_ |
| Bladder | 26.2 ± 17.94 | 69.58 ± 43.46 | 26.6 ± 6.73 | 54.76 ± 16.41 | 4.30 ± 0.95 | 10.07 ± 2.92 |
| Bone | 0.37 ± 0.06 | 0.63 ± 0.15 | 0.45 ± 0.11 | 0.81 ± 0.26 | 0.56 ± 0.10 | 0.96 ± 0.36 |
| Brain | 0.05 ± 0.01 | 0.37 ± 0.09 | 0.07 ± 0.03 | 0.57 ± 0.17 | 0.08 ± 0.01 | 0.67 ± 0.24 |
| Heart | 0.10 ± 0.03 | 0.34 ± 0.04 | 0.10 ± 0.04 | 0.41 ± 0.15 | 0.19 ± 0.04 | 0.56 ± 0.11 |
| Kidneys | 1.09 ± 0.24 | 2.20 ± 0.93 | 6.45 ± 2.40 | 11.33 ± 4.13 | 17.29 ± 2.12 | 30.46 ± 5.09 |
| Lacrimal glands | 0.15 ± 0.02 | 0.36 ± 0.07 | 0.32 ± 0.14 | 0.61 ± 0.35 | 1.26 ± 0.36 | 1.87 ± 0.62 |
| Liver | 0.12 ± 0.01 | 0.29 ± 0.07 | 0.09 ± 0.03 | 0.21 ± 0.07 | 0.14 ± 0.03 | 0.29 ± 0.11 |
| Muscle | 0.08 ± 0.02 | 0.25 ± 0.07 | 0.07 ± 0.01 | 0.25 ± 0.14 | 0.10 ± 0.03 | 0.30 ± 0.08 |
| Salivary glands | 0.11 ± 0.03 | 0.36 ± 0.24 | 0.24 ± 0.07 | 0.41 ± 0.12 | 0.93 ± 0.19 | 1.66 ± 0.39 |
| Spleen | 0.15 ± 0.04 | 0.47 ± 0.20 | 0.42 ± 0.05 | 1.54 ± 1.46 | 1.52 ± 0.44 | 3.06 ± 1.54 |
